# Multiscale bone remodeling in COVID-19: from osteoimmune signaling to structural and mechanical impairment

**DOI:** 10.3389/fbioe.2026.1812439

**Published:** 2026-07-21

**Authors:** G. Jiang, F. Buccino, L. M. Vergani

**Affiliations:** 1 Department of Mechanical Engineering (DMEC), Politecnico di Milano, Milan, Italy; 2 IRCCS Galeazzi-Sant’Ambrogio, Milan, Italy

**Keywords:** bone mechanobiology, bone microenvironment, COVID-19, lacunae, osteoimmunology

## Abstract

The skeletal consequences of COVID-19 have emerged as an important but still underrecognized component of post-viral systemic disease. Increasing clinical, experimental, and imaging-based evidence indicates that SARS-CoV-2 infection may impair bone health through multiscale mechanisms involving bone remodeling, osteoimmune signaling, vascular regulation, and mechanical adaptation. In this review, we summarize current evidence linking COVID-19 to skeletal deterioration, ranging from changes in bone mineral density, trabecular microarchitecture, and mechanical competence to osteocyte lacunar remodeling and altered bone-cell activity. We discuss both potential direct and indirect mechanisms by which SARS-CoV-2 may affect the skeletal system. Direct effects may involve viral infection-associated inflammatory and oxidative responses that promote osteoclastogenesis and bone resorption. Indirectly, SARS-CoV-2 may disturb bone homeostasis through ACE2 downregulation and renin–angiotensin system imbalance, systemic inflammation, endothelial dysfunction, glucocorticoid exposure, avascular necrosis, prolonged bed rest and mechanical unloading, vitamin D deficiency, and mineral dysregulation. Based on well-characterized mechanisms from other osteotropic viral diseases, we propose possible modes by which SARS-CoV-2 may affect the skeletal system. This review emphasizes key knowledge gaps, including the limited availability of longitudinal human data, the need for advanced imaging and multiscale mechanical modeling, and the potential role of host genetic susceptibility. Overall, COVID-19-related skeletal impairment should be considered a multifactorial and multiscale process. Integrated clinical, molecular, imaging, genetic, and biomechanical approaches will be essential to identify vulnerable individuals, monitor long-term skeletal outcomes, and develop targeted strategies to preserve bone health in COVID-19 survivors.

## 1 Introduction

Coronavirus disease 2019 (COVID-19), caused by the severe acute respiratory syndrome coronavirus 2 (SARS-CoV-2), has brought unprecedented challenges to global health (Wurm et al., 2024; Maison et al., 2025; Ioannidis et al., 2025). The virus enters human cells through binding of its spike (S) protein to the angiotensin-converting enzyme 2 (ACE2) receptor, leading to widespread damage not only to the respiratory system but also to multiple organs including the cardiovascular, renal, and immune systems (V'Kovski et al., 2020; Shang et al., 2020a; Shang et al., 2020b; Chang et al., 2022; Tereshchenko et al., 2022; Patone et al., 2021; Mahalingasivam et al., 2022; Hassler et al., 2021; Ivković et al., 2025; Sette and Crotty, 2021; Teijaro and Farber, 2021; Rodríguez et al., 2020). Since the World Health Organization declared COVID-19 a global pandemic in March 2020, over 767 million confirmed cases and more than 6.9 million deaths have been reported worldwide by June 2025, causing irreparable loss to society and healthcare systems.

**FIGURE 1:**
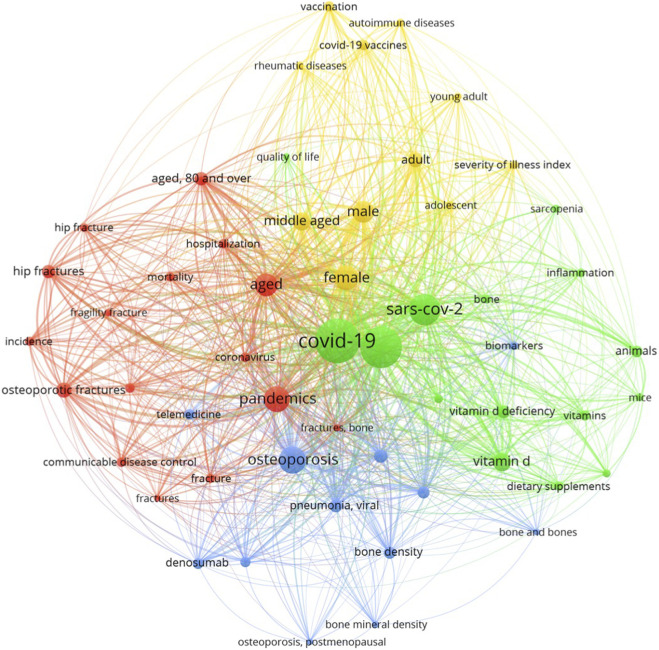
VOSviewer keyword co-occurrence map of COVID-19-related research. This network visualization illustrates the co-occurrence relationships among keywords in COVID-19-and bone- related literature. The bibliometric dataset was obtained from PubMed using the search terms “COVID-19” and “bone”, and the map was generated by the authors using VOSviewer 1.6.20^41^. Each node represents a keyword, with node size corresponding to its frequency and edge thickness representing co-occurrence strength. Colors indicate distinct clusters, reflecting thematic groupings within the research field.

While much attention has been focused on the pulmonary and cardiovascular consequences of SARS-CoV-2 infection, recent studies have begun to reveal its impact on the skeletal system, as summarized in the VOSviewer-based keyword co-occurrence map shown in Figure 1 (Van Eck and Waltman, 2010; Qiu et al., 2024; Karpiel et al., 2022; Sinha et al., 2022; da Silva et al., 2023; Awosanya et al., 2022). COVID-19 is now understood to exert both direct and indirect effects on bone health (Kerschan-Schindl et al., 2023; Alghamdi et al., 2024; Sapra et al., 2022; Tsourdi et al., 2022). On one hand, the infection induces a cytokine storm—characterized by elevated levels of interleukin-1 (IL-1), interleukin-6 (IL-6), and tumor necrosis factor-alpha (TNF-α)—that enhances osteoclast activation and accelerates bone resorption (Mehta et al., 2020; Fan et al., 2022). On the other hand, prolonged bed rest, glucocorticoid therapy, nutritional deficiencies, and systemic inflammation during and after infection may further disrupt bone homeostasis, particularly in individuals with preexisting conditions such as osteoporosis (Hayes et al., 2020; Mehri and Finsterer, 2024; Mei et al., 2020).

Osteoporosis (OP) is a systemic skeletal disorder marked by low bone mass and microarchitectural deterioration, resulting in increased fracture risk (Glaser and Kaplan, 1997). It is particularly concerning that emerging evidence suggests COVID-19 may worsen bone loss and increase fracture susceptibility in osteoporotic patients (Liu et al., 2022; Buc et al., 2023). Although several studies have discussed potential mechanisms linking COVID-19 to alter bone metabolism, direct clinical evidence remains limited (Sinha et al., 2021; Salvio et al., 2020; Lee et al., 2021). Moreover, specific data on how SARS-CoV-2 affects bone turnover and treatment responses in osteoporotic populations are lacking.

Despite growing recognition of SARS-CoV-2’s systemic manifestations, the long-term consequences on the skeletal system remain largely underexplored. Unlike pulmonary or cardiovascular sequelae, skeletal complications are often silent, slowly progressive, and easily overlooked (Chang et al., 2022; Qiu et al., 2024). However, mounting evidence suggests that COVID-19 may exert lasting effects on bone structure and turnover, particularly in vulnerable populations such as elderly individuals or those with preexisting osteoporosis (Mi et al., 2020). Clinical and systematic-review evidence has reported reduced bone mineral density, altered bone turnover markers, and musculoskeletal complications in patients during acute, post-acute, and long COVID-19 phases (Mi et al., 2020). In parallel, experimental studies using SARS-CoV-2-infected mouse and golden Syrian hamster models have demonstrated trabecular bone loss, increased osteoclast activity, and inflammatory bone remodeling (Awosanya et al., 2021; Qiao et al., 2022). More recently, large-scale synchrotron phase-contrast micro-CT combined with AI-based lacunar recognition in human femoral head trabecular bone revealed disease-associated osteocyte lacunar remodeling in both osteoporosis and post-COVID-19 bone, indicating that COVID-19-related skeletal impairment may involve microstructural alterations of the osteocyte mechanosensory network (Hosseini et al., 2026). These findings suggest that COVID-19-related skeletal alterations are not restricted to a single pathway, but may arise from the combined effects of systemic inflammation, osteoimmune dysregulation, altered bone turnover, vascular dysfunction, glucocorticoid exposure, vitamin D deficiency, mechanical unloading, and lacunar-level mechanobiological remodeling. However, the available evidence remains scattered across clinical observations, animal models, molecular studies, advanced imaging, and biomechanical investigations. This fragmentation highlights the need for an integrated review that connects biological mechanisms with structural and mechanical consequences of COVID-19-associated bone impairment.

In this review, we discuss current evidence on how COVID-19 affects bone health from both biomechanical and biological perspectives. By drawing on insights from clinical studies, imaging analyses, animal models, and analogous viral diseases, we highlight the urgent need to consider the skeletal system as a potential site of chronic post-COVID damage. Our goal is to raise awareness of these delayed but significant effects, and to call for long-term monitoring and multidisciplinary intervention strategies to preserve skeletal health in COVID-19 survivors.

## 2 Structural and mechanical alterations of bone following SARS-cov-2 infection

**FIGURE 2:**
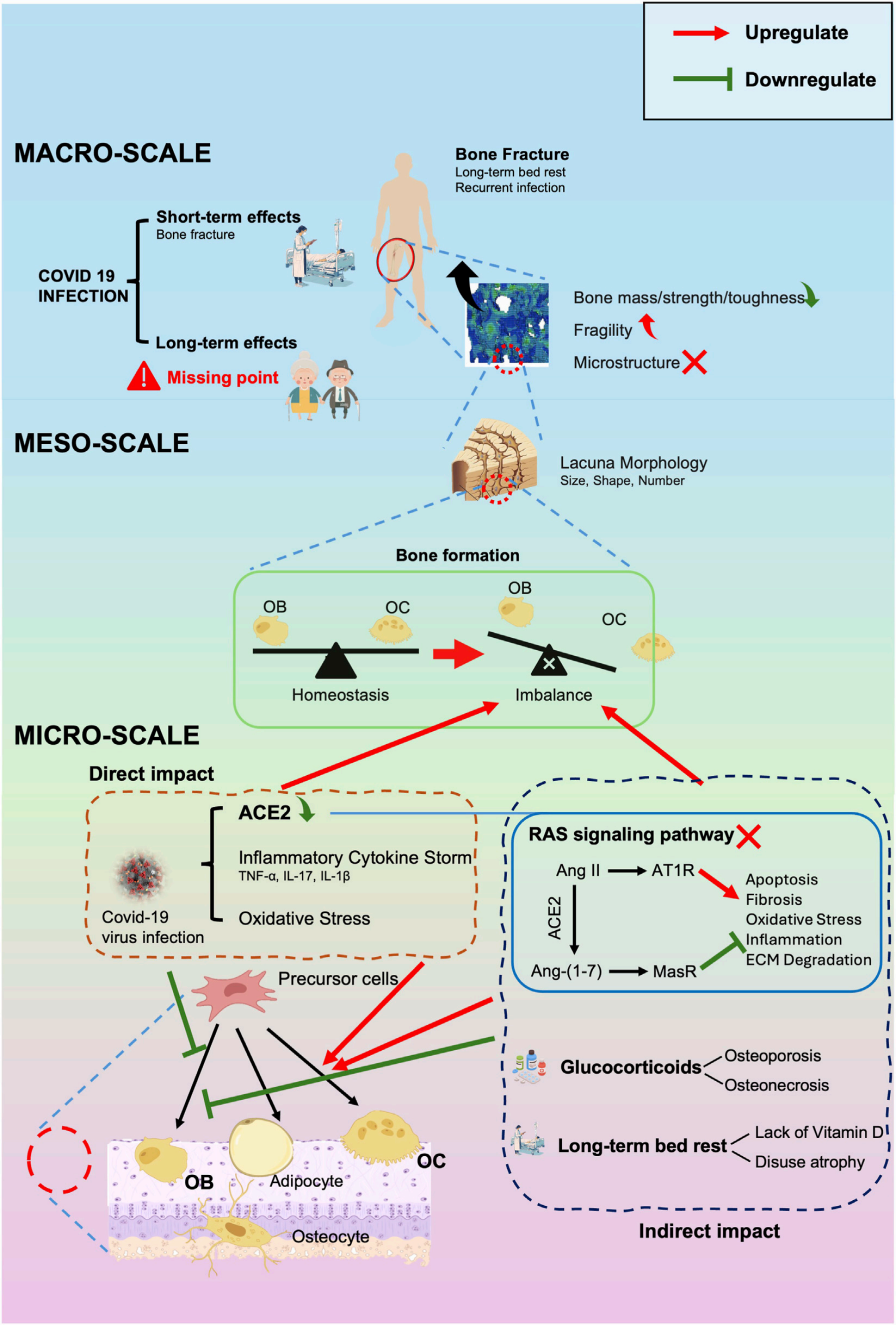
Potential mechanisms linking COVID-19 infection to skeletal deterioration. Illustration of the multiscale impact of COVID-19 on skeletal health. At the macro-scale, long-term bed rest and repeated infections increase fracture risk. At the meso-scale, bone mass, strength, and microarchitecture are affected. At the micro-scale, alterations in osteocyte lacunar morphology suggest disrupted mechanotransduction. Mechanistically, SARS-CoV-2 infection directly triggers inflammatory cytokine storms and oxidative stress via ACE2-mediated pathways, disrupting the balance between osteoblasts (OB) and osteoclasts (OC), thereby promoting osteoclastogenesis and bone-resorptive activity. The renin-angiotensin system (RAS) is also dysregulated, enhancing bone resorption and impairing formation. Additional indirect contributors include glucocorticoid therapy, vitamin D deficiency, and reduced mechanical loading, which collectively impair bone remodeling and promote fragility.

Bone is a mechanically responsive tissue whose structural integrity relies on homeostasis between resorption and formation, regulated by both biochemical and biomechanical factors. Emerging evidence suggests that SARS-CoV-2 infection disrupts this balance, not only through inflammatory and metabolic stressors but also via altered bone mechanobiology (Figure 2).

### 2.1 Macroscale structural and mechanical alterations in Post-COVID bone

Large-scale structural integrity of bone is central to fracture resistance and mechanical performance. In patients recovering from COVID-19, a growing body of evidence has revealed quantifiable deterioration in bone quality at the tissue and organ levels. These changes—encompassing bone mineral density loss, cortical thinning, and trabecular rarefaction—are detectable by clinical imaging and can be modeled through finite element analysis to estimate biomechanical competence (Haudenschild et al., 2023).

At the mesoscale, COVID-19 patients exhibit a marked decline in bone mineral density (BMD), cortical thickness, and trabecular connectivity (Zhang et al., 2024; Wang et al., 2024; Berktaş et al., 2022). These structural alterations are indicative of compromised biomechanical performance, including reduced load-bearing capacity and fracture resistance (Alghamdi et al., 2024). Finite element analysis developed by means of synchrotron scans further demonstrates diminished stiffness and toughness in both cortical and trabecular compartments, suggesting a diffuse degradation of mechanical competence across skeletal regions (Buc et al., 2023).

### 2.2 Microscale changes in osteocyte morphology and function

At the microscale, post-COVID-19 bone tissue exhibits morphometric disruption of the osteocyte network, a critical regulator of mechanosensing and bone homeostasis.

**FIGURE 3:**
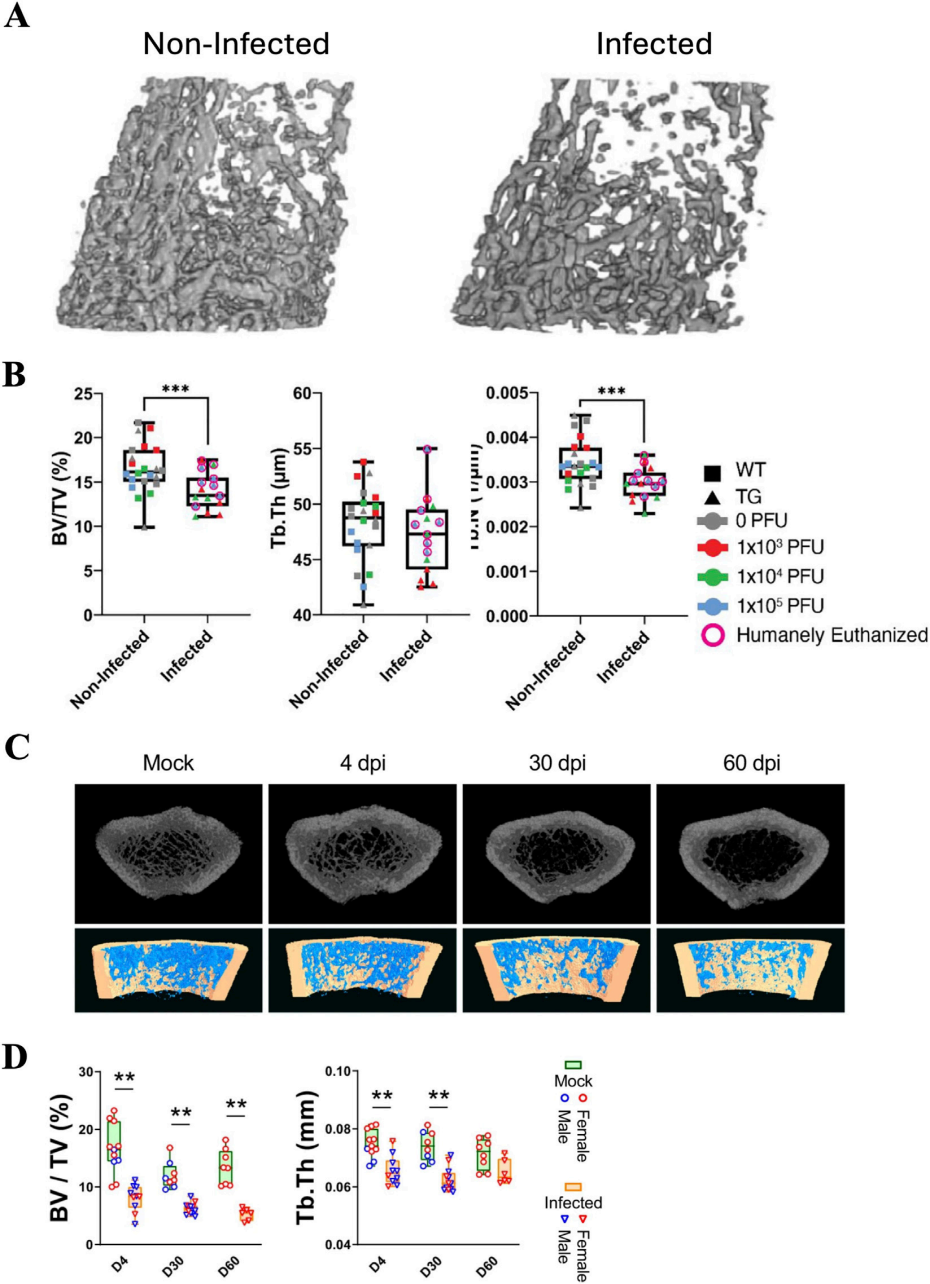
Effects of SARS-CoV-2 infection on femoral trabecular bone parameters in animal models **(A)** Representative 3D reconstructed micro-CT images show reduced trabecular density in femurs of SARS-CoV-2-infected mice compared to non-infected controls. **(B)** Quantitative analysis reveals significant decreases in bone volume fraction (BV/TV), trabecular thickness (Tb.Th), and trabecular number (Tb.N) following infection. (Awosanya et al., 2021). **(C)** Time-course micro-CT imaging at 4-, 30-, and 60-day post-infection (dpi) illustrates progressive deterioration of trabecular structure. **(D)** Bone morphometric data demonstrate sustained loss in BV/TV and Tb.Th in both male and female golden Syrian hamster model over time, indicating a long-lasting impact of SARS-CoV-2 infection on skeletal microarchitecture (Qiao et al., 2022).

Figures 3A–D demonstrates consistent reductions in BV/TV and Tb.Th in femoral trabecular bone in both the COVID-19 golden Syrian hamster and mouse models, whereas a significant decline in Tb.N was observed in the mouse model (Awosanya et al., 2021; Qiao et al., 2022).

**FIGURE 4:**
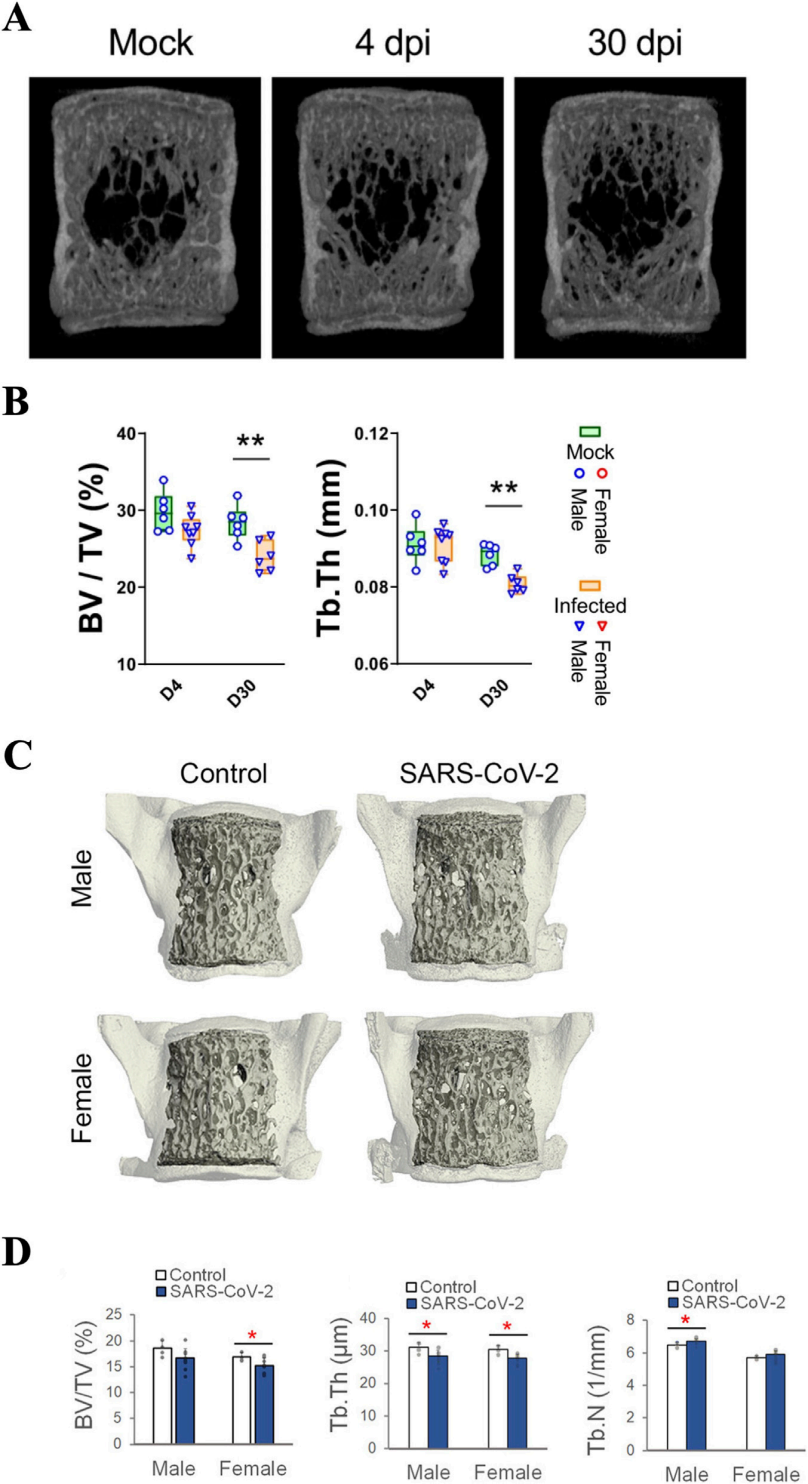
Effects of SARS-CoV-2 infection on vertebral trabecular bone parameters in animal models **(A)** Micro-CT images (top) and H&E-stained histological sections (bottom) show progressive bone loss and microarchitectural deterioration in vertebral trabeculae at 4- and 30-day post-infection (dpi) compared to mock controls. **(B)** Quantitative analysis indicates significant reductions in bone volume fraction (BV/TV) and trabecular thickness (Tb.Th) over time following infection (Qiao et al., 2022). **(C)** 3D reconstructed vertebral trabecular structures in male and female mice demonstrate sex-independent bone deterioration in the SARS-CoV-2-infected group. **(D)** Quantitative analysis in control and SARS-CoV-2–infected mice stratified by sex. Bar graphs show BV/TV decreased in both sexes, but significance was detected only in females; Tb.Th was significantly reduced in both sexes; and Tb.N increased in both sexes, but significance was detected only in males. (Haudenschild et al., 2023).

Figures 4A–D additionally shows that reduced BV/TV and Tb.Th were observed in the vertebral trabecular bone of both COVID-19 golden Syrian hamster and mouse models. Interestingly, an increase in Tb.N was detected only in the SARS-CoV-2-infected male mice (Haudenschild et al., 2023). Buccino et al. employed synchrotron-based, AI-assisted three-dimensional imaging to systematically characterize osteocyte lacunar alterations in human trabecular bone following COVID-19 infection, revealing a pronounced shift toward larger, rounder, and less oblate lacunae, together with increased lacunar volume and number density at the microscale. Importantly, despite relatively preserved meso-scale mechanical properties, these changes closely mirrored osteoporotic lacunar phenotypes and were associated with altered local stress distributions, elevated stress intensity factors, and impaired microcrack deflection (Buc et al., 2023). These changes are thought to reflect stress-induced remodeling, potentially driven by hypoxia, systemic inflammation, or altered interstitial fluid flow.

Osteocytes act as the principal mechanotransducers within the bone matrix, integrating mechanical strain with molecular signaling pathways such as Wnt/β-catenin and RANKL/OPG. Disruption of lacunar-canalicular architecture may obstruct fluid shear stress transmission and impair the osteocyte’s capacity to regulate local remodeling. Notably, such lacunar changes have also been observed in aging and primary osteoporosis, suggesting a parallel pathological mechanism.

A comparative overview is provided in Table 1, summarizing osteocyte alterations in healthy, aging, primary osteoporosis, and post-COVID-19.

**TABLE 1:** Comparative osteocyte lacunar alterations in aging, primary osteoporosis, and post-COVID-19 bone.

Condition	Lacunar size	Shape index (sphericity)	Lacunar density	Functional implication
Healthy adult	Baseline	Elongated (low)	Normal	Efficient mechanosensing
Aging (Hering et al., 2024)	↑	↑	↓	Mechanosensing decline
Primary osteoporosis (Buc et al., 2023)	↑	↑	↑	Osteocyte apoptosis, canalicular rarefaction
Post-COVID-19 (Buc et al., 2023; Haudenschild et al., 2023)	↑	↑	↑	Inflammatory remodeling, fluid shear disturbance

As summarized in Table 1, post-COVID-19 osteocyte morphology exhibits a distinct signature of increased size and density, differing from the apoptotic rarefaction seen in primary osteoporosis. These alterations suggest a remodeling-driven adaptation rather than passive degeneration.

### 2.3 Imaging modalities and quantification of bone microarchitecture

Recent advances in imaging and morphometric analysis have enabled the visualization of osteocyte lacunar changes with increasing resolution and mechanistic insight. Traditional histomorphometry using 2D undecalcified bone sections has identified lacunar hypertrophy in iliac crest biopsies from COVID-19 patients. However, newer modalities such as synchrotron-based micro-CT allow 3D reconstructions of lacunar-canalicular networks with submicron precision, permitting assessment of connectivity, anisotropy, and spatial organization (Nango et al., 2013; Wittig et al., 2019). In addition, quantitative backscattered electron imaging (qBEI) has been employed to assess bone matrix mineralization surrounding lacunae, revealing abnormal peri-lacunar demineralization zones in aging and osteoporosis models (Blouin et al., 2024; Sutton-Smith et al., 2008; Hartmann et al., 2021).

Beyond morphology, emerging techniques such as Brillouin microscopy and nanoscale AFM-based elastography offer insights into the local viscoelastic environment of osteocytes and their lacunae (Alunni et al., 2022; Yvanoff and Willaert, 2022) (Table 2). In animal models, these methods have demonstrated altered pericellular matrix stiffness after systemic inflammation, implying that osteocytes in post-COVID-19 bone may also operate within a mechanically dysregulated microenvironment.

**TABLE 2:** Imaging and characterization techniques to evaluate osteocyte morphology, considered parameters and related limitations.

Technique	Resolution	Key parameters assessed	Limitations
Histology	∼1–2 μm	Area, shape, density	Lacks 3D topology
Micro-CT/nano-CT	∼1–5 μm/<1 μm	Volume, anisotropy, spatial distribution	Radiation dose, limited trabecular resolution
Synchrotron CT	<0.5 μm	Canalicular mapping, ultra-fine structures	Limited access, complex image processing
qBEI (SEM-based)	∼0.5 μm	Lacunar margin mineral density, osteolysis zones	Surface prep needed; no 3D
Brillouin microscopy	∼0.3 μm	Local stiffness, viscoelastic properties	Signal-to-noise, low adoption in hard tissue
AFM-based nanoindentation	∼0.1 μm	Peri-lacunar modulus, stiffness gradients	Surface-limited, requires exposed bone sections

Table 2 summarizes the key imaging and characterization techniques used to evaluate osteocyte morphology and mechanical context across bone loss conditions.

### 2.4 Temporal dynamics of bone impairment after SARS-cov-2 infection

Unlike the rapid immune recovery observed in many COVID-19 survivors, bone is a metabolically active yet slowly remodeling tissue, and its response to systemic perturbation often lags behind that of other organ systems (Bhor et al., 2024). Early bone loss is marked by enhanced resorption and suppressed formation, while structural recovery may lag for months or remain incomplete. Longitudinal studies in both clinical and experimental settings have begun to illuminate the timeline of post-infectious skeletal deterioration.

In the acute phase (within the first 2–4 weeks post-infection), patients exhibit elevated serum markers of bone resorption (CTX, TRAP5b), suppressed bone formation indices (P1NP, osteocalcin, etc.), and, in severe cases, microarchitectural disruption detectable by HR-pQCT or vertebral morphometry (Ma et al., 2023; Queiroz-Junior et al., 2023). These changes are largely attributed to systemic inflammation, hypercortisolemia, and mechanical unloading.

In the subacute and chronic phases (3 months and beyond), longitudinal imaging studies have shown that while biochemical markers may normalize, structural deterioration often persists. A multicenter cohort from Russia reported that vertebral BMD remained significantly lower at 6 months post-recovery, particularly in ICU-treated individuals (Petraikin et al., 2025). Animal models echo this, demonstrating persistent alterations in trabecular number, osteocyte density, and peri-lacunar remodeling as far as 12 weeks post-infection (Eltobgy et al., 2025).

Notably, such prolonged remodeling deficits resemble patterns observed in chronic inflammatory conditions such as rheumatoid arthritis or post-HIV osteoporosis, suggesting a shared “inflammation-aging” mechanism (Ebeling et al., 2022; Schwenke et al., 2023; Madanhire et al., 2024; Srivastava et al., 2022). These findings support the need for long-term monitoring of skeletal health in COVID-19 survivors, even in the absence of overt symptoms.

## 3 Direct Effects of SARS-coV-2 on the Skeletal System

### 3.1 Direct interaction between the virus and bone cells

While systemic inflammation is a well-known driver of COVID-19 complications, emerging evidence points to the skeletal system as a potential site of direct viral targeting (Florencio and Fernández-de-Las-Peñas, 2022; Effenberger et al., 2020). The virus gains cellular entry via binding to angiotensin-converting enzyme 2 (ACE2), facilitated by TMPRSS2-mediated spike protein priming (Cui et al., 2022). Bone-resident cells—including osteoblasts, osteoclasts, and their progenitors—express varying levels of the SARS-CoV-2 entry factors ACE2 and TMPRSS2. This raises the possibility that SARS-CoV-2 may infect and impair bone cells directly, independent of systemic cytokine effects.

**FIGURE 5:**
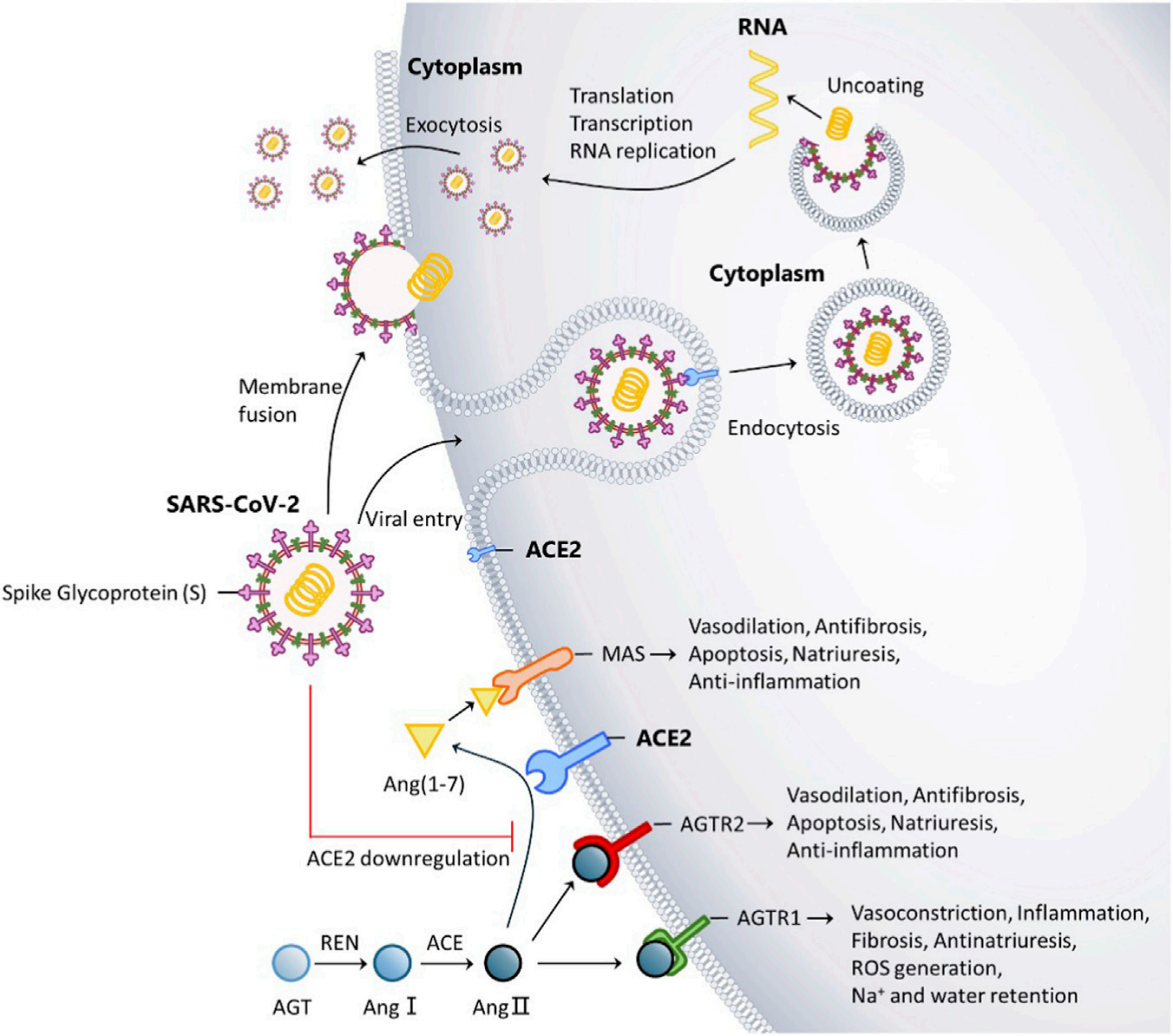
SARS-CoV-2 cellular entry mechanism, downstream signaling pathways, and Bone turnover marker related to COVID-19. SARS-CoV-2 enters human host cells via binding of its spike (S) glycoprotein to the ACE2 receptor, leading to membrane fusion or endocytosis, and subsequent release and replication of viral RNA. The infection causes downregulation of ACE2, disrupting the balance of the renin–angiotensin system (RAS). This shift favors Angiotensin II (Ang II) signaling through the AGTR1 receptor, promoting vasoconstriction, inflammation, oxidative stress, and fibrosis. Simultaneously, protective pathways mediated by Ang-(1–7) and MAS/AGTR2 are suppressed.

Several transcriptomic studies, including single-cell RNA sequencing of human bone marrow and murine calvaria, have confirmed low-to-moderate ACE2 expression in osteoclasts, and chondrocytes, with TMPRSS2 co-expression most prominent in osteoblast progenitors (Achom et al., 2022) (Figure 5). Immunohistochemistry of femoral bone biopsies from COVID-19 autopsies has also revealed spike protein staining in endosteal cells and hypertrophic chondrocytes, supporting *in situ* viral tropism (Uranaka et al., 2020).

### 3.2 SARS-CoV-2-induced bone metabolism dysregulation

A hallmark of COVID-19 pathophysiology is the cytokine storm, characterized by elevated circulating levels of IL-6, TNF-α, and IL-1β (Coperchini et al., 2021; Jiang et al., 2022). These pro-inflammatory cytokines exert profound effects on bone remodeling by shifting the balance toward net resorption (Terkawi et al., 2022).

Mechanistically, IL-1β and TNF-α enhance RANKL expression and suppress osteoprotegerin in both stromal and osteoblastic cells, amplifying osteoclastogenesis (Suárez et al., 2000; Le et al., 2022; Strober, 2022). IL-6 further promotes osteoclast precursor differentiation via JAK/STAT3 activation, while simultaneously inhibiting osteoblast function (Ru et al., 2023). Collectively, these signaling cascades converge on the NF-κB axis, a master regulator of inflammatory bone loss (Yao et al., 2021).

In murine models of SARS-CoV-2 or spike protein exposure, trabecular bone loss is accompanied by marrow infiltration of CD11b^hi^ inflammatory monocytes, mirroring changes seen in inflammatory arthritis (Privratsky et al., 2022). This inflammatory remodeling phenotype resembles other systemic conditions, including: rheumatoid arthritis: IL-6 and TNF-driven RANKL upregulation leads to joint erosion; HIV: chronic immune activation leads to low BMD via similar cytokine-mediated mechanisms; severe osteoporosis: seen in ICU patients with systemic inflammation and immobilization (Cai et al., 2022; Liu et al., 2024; Shen et al., 2021; Soldado-Folgado et al., 2022).

Thus, SARS-CoV-2 infection triggers a state of immuno-skeletal uncoupling, wherein heightened immune signaling overrides normal bone remodeling control mechanisms. And this effect may persist even after viral clearance, contributing to long-term skeletal deficits in COVID-19 survivors.

### 3.3 SARS-CoV-2 disrupts the bone marrow niche

The bone marrow microenvironment is a highly structured niche that hosts and regulates hematopoietic and mesenchymal stem cells (HSCs and MSCs), both of which are crucial for bone homeostasis and immune competence (Zheng et al., 2022; Bandyopadhyay et al., 2024). SARS-CoV-2 exerts profound effects on this ecosystem, through both direct cellular invasion and systemic inflammatory remodeling.

In COVID-19 patients, bone marrow hematopoiesis often shifts toward the myeloid lineage, with elevated granulopoiesis and monocytosis observed in both clinical and experimental studies (Elahi, 2022). This pro-inflammatory microenvironment sustains osteoclastogenesis via excess RANKL and M-CSF activation (Melano et al., 2024). Moreover, autopsy studies of COVID-19 victims have revealed hypocellular marrow, fatty replacement, and altered vascular niches, all of which may compromise stem cell viability and osteogenic capacity (Greuel et al., 2021; Marques-Maggio et al., 2023).

These evidences support the notion that SARS-CoV-2 induces a niche exhaustion phenotype, whereby chronic inflammation and direct viral interference compromise the cellular foundation required for skeletal maintenance and regeneration. Given the regenerative role of the marrow niche in fracture repair and adaptation, its impairment could contribute to long-term bone fragility even in recovered patients.

### 3.4 Disrupted cellular signaling pathways in COVID-19-associated bone loss

SARS-CoV-2 infection disturbs bone homeostasis by engaging multiple intracellular signaling cascades that regulate osteogenesis and bone resorption (Zhang J. et al., 2025; Galliera et al., 2024). These pathways act downstream of both direct viral entry and inflammatory stimuli, constituting a complex mechanochemical signaling web that reprograms skeletal cell fate.

- NF-κB Pathway

The NF-κB signaling cascade is a central mediator of inflammation-induced bone loss (Park et al., 2025). Cytokines such as TNF-α and IL-1β activate NF-κB in both osteoblast precursors and immune cells, leading to increased RANKL transcription, reduced OPG production, and enhanced osteoclast differentiation (Teng et al., 2021). NF-κB also inhibits osteoblastogenesis by suppressing BMP/Smad signaling, further tipping the balance toward resorption (Boyce et al., 2023).

b. JAK/STAT3 Pathway

IL-6 signals via the JAK/STAT3 axis, which promotes osteoclastogenic gene expression and inhibits osteoblast maturation (Zhou et al., 2017; Ma et al., 2018). Elevated STAT3 phosphorylation has been observed in MSCs exposed to COVID-19 plasma. Importantly, blockade of this pathway with tocilizumab (anti-IL-6R) has shown partial reversal of osteogenic inhibition *in vitro*, suggesting therapeutic relevance.

c. Wnt/β-Catenin Pathway Suppression

The Wnt/β-catenin pathway is a crucial driver of osteoblast proliferation, differentiation, and survival (Almeida et al., 2012; Oh et al., 2022). Inflammatory stress during COVID-19 upregulates antagonists such as sclerostin (SOST) and DKK1, which inhibit Wnt signaling (Vallée et al., 2021). Murine models of systemic inflammation show decreased nuclear β-catenin and RUNX2 activity in bone tissues after SARS-CoV-2 spike protein exposure (Nong et al., 2024). These findings implicate Wnt suppression in the impaired bone formation observed post-infection.

d. Oxidative Stress and SIRT1 Disruption

SARS-CoV-2-induced oxidative stress depletes cellular NAD^+^ pools and inhibits SIRT1, a histone deacetylase involved in osteoblast survival, mitochondrial homeostasis, and anti-inflammatory signaling (Bhasin et al., 2023; Zhang S. et al., 2022). Loss of SIRT1 promotes senescence and adipogenic drift in MSCs, further impairing regenerative potential (Izadpanah et al., 2023). Activation of SIRT1 by agents such as resveratrol or SRT2104 has shown promise in restoring osteogenic capacity in oxidative models (Lu et al., 2022; Zhang J. et al., 2022).

### 3.5 Insights from viral bone pathologies: Comparative mechanisms

Although COVID-19 has brought renewed attention to the skeletal consequences of viral infections, it is not the first virus associated with bone loss. Comparative analysis with other viral diseases reveals convergent pathological mechanisms, including chronic inflammation, immune dysregulation, and direct disruption of bone marrow niches.

- Human Immunodeficiency Virus (HIV)

Up to 60% of HIV-infected individuals show evidence of osteopenia or osteoporosis (Blanco et al., 2022; Biver, 2022). Mechanisms include chronic immune activation, elevated TNF-α, direct infection of mesenchymal stem cells, and the effects of antiretroviral therapy (Olali et al., 2022). HIV inhibits Wnt/β-catenin signaling and enhances NF-κB-mediated osteoclastogenesis—mechanistically paralleling COVID-19-induced pathways.

b. Hepatitis C Virus (HCV)

Chronic HCV infection has been linked to increased fracture risk and reduced BMD, especially in cirrhotic patients (Weinstein et al., 2025). Mechanisms include: elevated IL-6 and oxidative stress levels; hepatic dysfunction leading to low vitamin D and IGF-1; upregulation of SOST and suppression of osteoblast activity; animal models show suppressed RUNX2 and increased RANKL expression, overlapping with post-COVID observations (Temesgen et al., 2025).

c. Influenza and Other Acute RNA Viruses

Severe influenza infection, especially with H1N1 strains, has been linked to transient osteopenia in children and adults (Lorente et al., 2021). Mechanisms include acute inflammatory cytokinemia, corticosteroid use, and immobilization. These acute-onset, self-limited bone losses resemble the early phase of COVID-19, though COVID more frequently triggers long-lasting bone remodeling impairment.

d. Cytomegalovirus (CMV) and Epstein–Barr Virus (EBV)

These DNA viruses can infiltrate bone marrow and induce myeloid skewing, stromal cell depletion, and vascular niche remodeling. CMV infection impairs osteoblast differentiation, while EBV is linked to marrow fibrosis in chronic infections (Sakai et al., 2021; Soldan and Lieberman, 2022). These niche-level disruptions are highly relevant to SARS-CoV-2-induced marrow changes.

e. Dengue and Zika Viruses

Though classically known for vascular tropism, both viruses show affinity for skeletal progenitors. Zika virus infects chondrocytes and impairs endochondral ossification in fetal models. Dengue has been associated with increased osteoclast activity via RANKL overexpression (Zhang J. et al., 2025).

These findings highlight that virus-induced alterations in bone and immune interactions extend beyond COVID-19 and represent a broader, underexplored category of post-viral skeletal complications. This suggests that antiviral treatment alone may not fully address the skeletal consequences, and that additional therapies targeting inflammation, oxidative damage, and disrupted bone remodeling pathways should be considered.

## 4 Indirect effects of SARS-cov-2 on the skeletal system

Skeletal impairment after COVID-19 is likely multifactorial and may occur even in the absence of direct viral infection of bone-resident cells. SARS-CoV-2 infection can reshape the systemic environment through ACE2 downregulation, renin–angiotensin system imbalance, persistent inflammation, endothelial dysfunction, hypercoagulability, therapeutic glucocorticoid exposure, mechanical unloading, and altered mineral metabolism. These factors do not act independently; rather, they may converge on common bone-remodeling pathways by enhancing osteoclastogenesis, suppressing osteoblast differentiation, impairing osteocyte mechanotransduction, and reducing the capacity for structural repair.

### 4.1 Dysregulation of the renin–angiotensin system (RAS)

Under physiological conditions, ACE2 converts angiotensin II (Ang II) into angiotensin-(1–7) [Ang-(1–7)], thereby limiting the vasoconstrictive, pro-inflammatory, and pro-oxidative effects of the classical ACE/Ang II/AT1R pathway while preserving the counter-regulatory, vasodilatory, and tissue-protective arm of the system. Because SARS-CoV-2 binds to ACE2 and promotes its internalization and functional downregulation, infection may shift the balance toward Ang II dominance and reduce Ang-(1–7)-mediated protection, with consequences that extend beyond the lung and vasculature to the bone microenvironment (Figure 2) (Bandeira et al., 2022). In skeletal tissue, excess Ang II has been associated with endothelial dysfunction, reduced microvascular perfusion, oxidative stress, and inflammatory signaling, all of which can impair osteoblast differentiation and favor osteoclastogenesis, thereby uncoupling bone remodeling (Beyerstedt et al., 2021). Conversely, ACE2 typically promotes the formation of angiotensin-(1–7), which exerts anti-inflammatory and anti-resorptive effects Conversely, loss of ACE2 activity may weaken local anti-resorptive and pro-angiogenic signaling, which is particularly relevant for bone regions that depend on a finely regulated blood supply, such as the femoral head and subchondral bone (Chhabra et al., 2025).

Therefore, SARS-CoV-2-mediated RAS imbalance may contribute to post-COVID bone fragility not only through systemic hemodynamic effects, but also through direct perturbation of the skeletal niche and its vascular support.

### 4.2 Physical inactivity and mechanical unloading

Hospitalized COVID-19 patients, particularly those requiring intensive care, often experience prolonged periods of immobility. This disuse state leads to mechanical unloading of the skeleton, a well-known stimulus for bone resorption (Tang, 2022). Since bone remodeling is tightly regulated by mechanical forces, unloading reduces osteogenic signaling and promotes a net catabolic state characterized by lower bone formation and increased resorption. Studies in both human and animal models have demonstrated that as little as one to 2 weeks of reduced mechanical loading can lead to significant losses in bone mass and strength (Liu et al., 2025). Over time, these changes can lead to trabecular rarefaction, cortical thinning, and delayed remodeling, thereby increasing fragility risk.

### 4.3 Effects of medications used in COVID-19 treatment

Corticosteroids remain a mainstay in the treatment of severe COVID-19 due to their potent anti-inflammatory effects. However, chronic or high-dose corticosteroid therapy is a well-established risk factor for secondary osteoporosis (Li et al., 2024; Fu et al., 2025). These drugs impair osteoblastogenesis, promote osteoblast and osteocyte apoptosis, and enhance osteoclast survival, leading to net bone resorption (Qiao et al., 2023). At the same time, the pandemic-associated disruption of osteoporosis management has led to delays in bone-protective therapies and reduced access to routine assessment, potentially compounding treatment-related bone loss. Therefore, medication-related skeletal effects in COVID-19 should be considered not only as an adverse drug consequence, but also as part of the broader post-COVID burden on bone health.

### 4.4 Avascular necrosis

Avascular necrosis (AVN), also known as osteonecrosis, may represent an important downstream skeletal consequence reported in COVID-19 survivors, particularly in the femoral head (Zahed et al., 2025). Although corticosteroid exposure remains the most widely recognized precipitating factor, emerging clinical evidence suggests that AVN after COVID-19 cannot be attributed to steroids alone (Migliorini et al., 2024). Systematic reviews have reported a temporal association between SARS-CoV-2 infection and subsequent AVN, with lesions most often involving the hip but also reported in the knee, shoulder, spine, and sacrum, and symptom onset occurring within weeks to a few months after infection (Sulewski et al., 2021). COVID-19-related endothelial dysfunction, hypercoagulability, microthrombosis, and inflammatory cytokine signaling may compromise the microcirculation of subchondral bone, thereby creating a hypoxic environment that favors osteocyte death, impaired angiogenesis and local remodeling, and ultimately weaken the trabecular structure (Veyre et al., 2020). Recent studies also suggest that host genetic factors may influence individual susceptibility to post-COVID-19 AVN, further supporting a multifactorial interaction between vascular dysfunction, inflammation, coagulation abnormalities, and genetic predisposition (Fouda et al., 2024; Foudaa et al., 2025). Therefore, early recognition of persistent hip, groin, shoulder, or knee pain in COVID-19 survivors, especially in those exposed to systemic corticosteroids or severe inflammatory disease, may be important for timely imaging-based diagnosis and joint-preserving intervention.

### 4.5 Vitamin D deficiency and mineral dysregulation

Vitamin D plays a central role in calcium-phosphate metabolism and bone health. COVID-19 patients frequently exhibit hypovitaminosis D, which may result from reduced sun exposure during lockdowns, malnutrition, or systemic inflammation (Thacher, 2021; Pereira et al., 2020). Deficiency in vitamin D leads to impaired intestinal calcium absorption and secondary hyperparathyroidism, both of which contribute to bone demineralization.

## 5 Discussion

### 5.1 Interdisciplinary approaches advance understanding of bone pathophysiology

The successful integration of biology and physics has significantly accelerated the development of quantitative strategies to address complex biological questions. In the context of bone research, this cross-disciplinary synergy is particularly relevant for dissecting how mechanical cues and inflammatory signals intersect during COVID-19–associated skeletal complications. Accumulating evidence suggests that developing tissues—including bone—exhibit dynamic viscoelastic properties, and that external mechanical forces such as compression, tension, shear, and confinement serve as principal drivers of tissue remodeling and cell fate specification (Gao et al., 2023; Lee et al., 2020). These forces act in concert with biochemical signals to shape bone morphology and regulate bone cell function (Zhang Q. et al., 2025). However, the nature of these interactions and their regulatory principles remain incompletely understood.

A key future direction involves the development of integrated platforms that can simultaneously measure and manipulate mechanical stimuli while tracking changes in cellular states in real time. Emerging imaging-based methods—especially those using genetically encoded tension sensors coupled with lineage tracing or cell fate reporters—will be particularly powerful (Yang et al., 2025; Shang et al., 2026). In parallel, organoid-based systems provide experimentally tractable platforms with controllable boundary conditions to unravel causality in mechano-chemical feedback loops (Makkieh et al., 2025). Advances in bioprinting and microfabrication are also enabling more realistic reconstruction of bone microenvironments, supporting improved modeling of mechanobiological complexity (Bhattacharyya and Noh, 2025).

### 5.2 Bridging scales: from molecules to tissues

**FIGURE 6:**
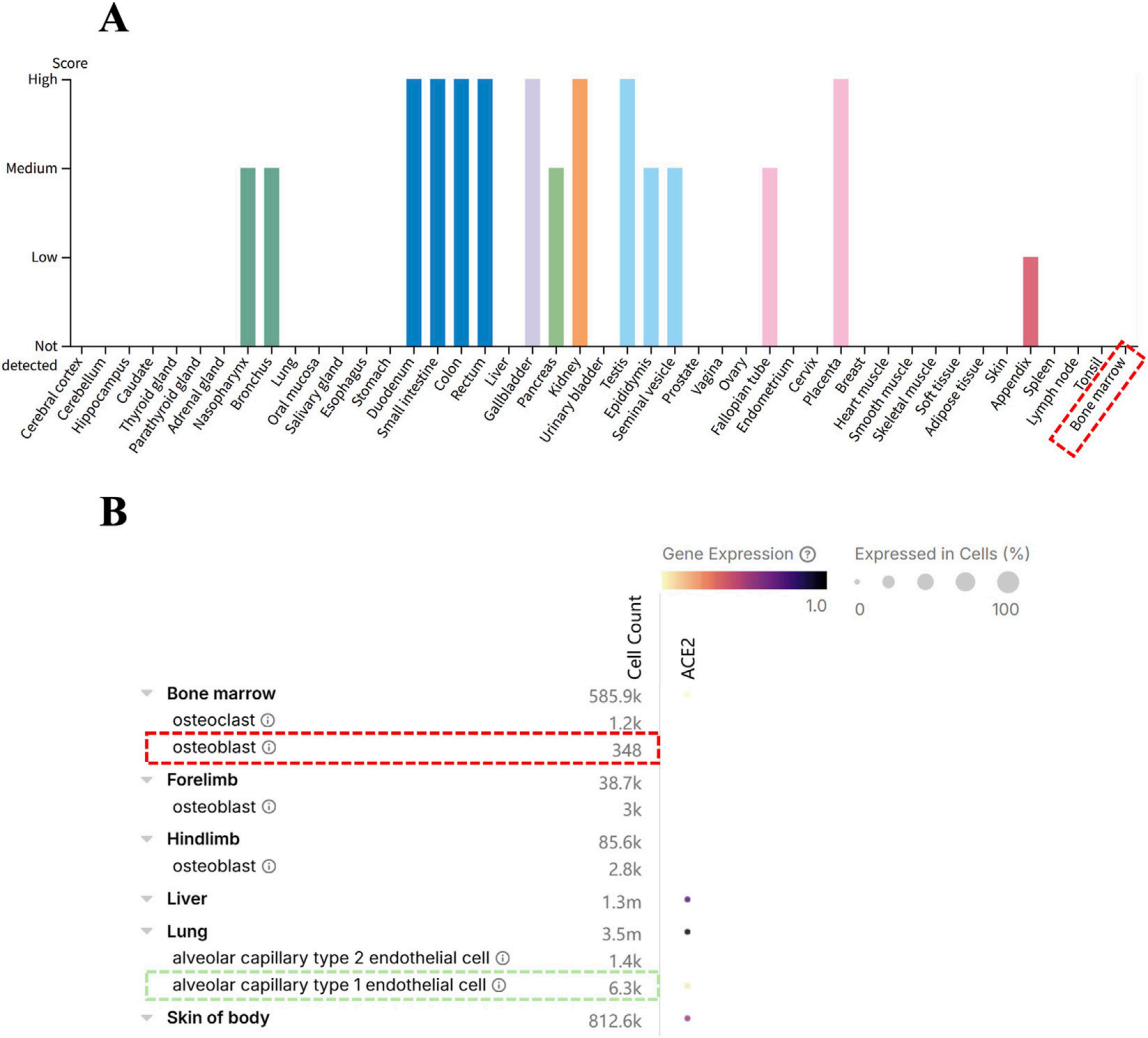
Low expression of ACE2 in osteoblasts suggests indirect skeletal effects of COVID-19 **(A)** Tissue-wide RNA and protein expression profiles of ACE2 demonstrate high expression in gastrointestinal and respiratory tissues but relatively low expression in bone marrow and lymphoid tissues **(B)** Single-cell RNA sequencing data reveal minimal ACE2 expression in osteoblasts and osteoclasts within the bone marrow, in contrast to higher expression in alveolar epithelial cells.

Although ACE2 has been identified as the main entry receptor for SARS-CoV-2 in various tissues, its expression in bone-forming cells appears to be extremely low (Andriankaja et al., 2025; Huang et al., 2026; Qi et al., 2026; Cao et al., 2026). As shown in recent single-cell transcriptomic datasets, ACE2 is barely detectable in osteoblasts, suggesting that the virus is unlikely to exert direct effects on these cells (Figure 6). This observation aligns with the hypothesis that the bone-related complications observed in COVID-19 patients may be driven primarily by indirect mechanisms. Such mechanisms include systemic inflammation, cytokine storms, dysregulation of the RAS pathway, and treatment-related factors (e.g., corticosteroids or immobilization), which together can disrupt bone remodeling and exacerbate bone fragility (Tang et al., 2025; Yu et al., 2026). Therefore, instead of a direct viral invasion of skeletal cells, it is the altered systemic milieu induced by COVID-19 that likely contributes to the observed skeletal manifestations (Zhang J. et al., 2025).

Another exciting Frontier lies in the development of approaches that bridge multiple biological scales. From a biophysical perspective, bone tissues display emergent mechanical properties, where molecular-level alterations—such as cytoskeletal rearrangements, adhesion dynamics, or signaling fluctuations—can reshape macroscopic tissue behavior (Buc et al., 2023; Yvanoff and Willaert, 2022). Bridging this gap demands real-time force quantification across entire bone structures, ideally using integrated *in vivo* force sensors and advanced imaging modalities.

Moreover, machine learning–assisted image segmentation and inference tools offer promising avenues to improve quantitative readouts of force distribution and mechanical deformation (Buc et al., 2023; Neijhoft et al., 2026). Time scale considerations are also critical: while methods exist to measure the elasticity of cells and tissues, viscous responses are often overlooked, despite their relevance in biological systems where force generation and transduction may occur on much slower time frames than typical mechanical testing captures. Longitudinal monitoring—spanning seconds to months—is essential for capturing how short-term mechanical responses translate into long-term developmental or pathological outcomes, especially in contexts such as bone remodeling during post-viral inflammation.

### 5.3 Modeling and simulation: A predictive toolbox

Alongside experimental advances, theoretical modeling and computational simulations provide invaluable frameworks for predicting how COVID-19–related disturbances propagate through skeletal systems. As the capacity to measure force-related parameters expands, it becomes increasingly challenging to isolate individual contributions experimentally (Ateeq et al., 2025; Brandenburg Dos Santos et al., 2026). Modeling thus becomes a necessary tool to explore large parametric spaces and to integrate molecular, cellular, and tissue-scale inputs into unified simulations.

Cell-based models—such as vertex, Potts, or self-propelled Voronoi frameworks—have already proven effective in exploring cell mechanics, morphodynamics, and transitions between flow-like and jammed states (Comlekoglu et al., 2025; Bi et al., 2016; Cowley et al., 2025). However, further refinements are needed to better reflect the dynamic complexity of bone microarchitecture, as well as to capture signal feedback, cell lineage transitions, and inflammation-mediated remodeling. Coupling these models with emerging experimental systems could yield powerful insights into how COVID-19 perturbs homeostatic bone remodeling pathways.

## 6 Conclusion and outlook

The COVID-19 pandemic, caused by SARS-CoV-2, has profoundly affected multiple organ systems, with increasing evidence pointing to the skeletal system as a previously underrecognized but biologically significant target. Our review synthesizes current biomechanical, cellular, and molecular evidence, underscoring that SARS-CoV-2 infection, either through direct viral entry or indirect systemic effects, can impair bone homeostasis, compromise structural integrity, and disrupt osteoimmune signaling.

While acute skeletal manifestations have been demonstrated in both clinical and experimental settings, the long-term consequences of COVID-19 on bone health remain largely underexplored. Given the prevalence of long COVID and post-acute sequelae, understanding whether and how SARS-CoV-2 induces lasting changes in bone remodeling, bone quality, and fracture risk is of paramount importance, particularly for aging populations or patients with pre-existing metabolic bone diseases.

Skeletal fragility may emerge not only from the inflammatory and hormonal disturbances associated with the infection but also from mechanical disuse, prolonged hospitalization, corticosteroid therapy, and disrupted calcium-phosphate metabolism. These multifactorial risks call for integrative studies that combine longitudinal clinical imaging, biomarker profiling, and mechanobiological modeling to dissect the persistence and reversibility of COVID-induced skeletal alterations.

Looking forward, future research may focus on multiple aspects. First, understanding the trajectory of skeletal changes in post-COVID patients requires well-designed long-term observational studies. These should include repeated assessments of bone mineral density (BMD), trabecular microarchitecture, cortical thickness, and fracture risk across various time points post-infection (e.g., 3, 6, 12, 24 months). Ideally, cohorts would be stratified by age, sex, pre-existing bone conditions, and COVID severity. Advanced imaging tools such as high-resolution peripheral quantitative computed tomography (HR-pQCT) and finite element modeling (FEM) could provide mechanistic insights into structural integrity loss and recovery. This would help distinguish between transient versus persistent bone alterations and identify individuals at highest risk for skeletal fragility. Meantime, investigating the contribution of host genetic susceptibility to COVID-19-associated skeletal impairment may also be an interesting direction. Inter-individual differences in bone loss, fracture risk after SARS-CoV-2 infection may partly reflect genetic variation in pathways involved in viral entry, immune activation, and bone remodeling. SNPs in ACE2 and other renin–angiotensin system-related genes may influence tissue susceptibility to SARS-CoV-2-associated RAS imbalance, whereas variants in cytokine-related genes may modulate the intensity and duration of inflammatory osteoclastogenic responses. However, direct evidence linking specific SNPs to post-COVID bone loss or osteonecrosis remains scarce. Future studies combining longitudinal skeletal phenotyping, bone turnover biomarkers, imaging-based microarchitectural analysis, and genome-wide or candidate-gene association approaches are needed to determine whether genetic variants can identify individuals at higher risk of COVID-19-related skeletal fragility. Additionally, preclinical studies have shown sex-specific differences in bone response to COVID-19, possibly due to hormonal modulation of ACE2 expression, immune responses, and bone turnover dynamics. Similarly, older adults, already predisposed to bone loss, may experience exacerbated effects due to comorbidities, inactivity, or polypharmacy during infection. Thus, future studies should perform stratified analyses to identify vulnerable subgroups who may benefit most from early screening or preventive intervention. Understanding these demographic nuances can also inform personalized treatment strategies. Finally, based on the multifactorial nature of COVID-induced bone fragility, an effective clinical response may involve multi-modal therapies: rehabilitation and mechanical loading; anti-resorptive therapy; anabolic agents; anti-inflammatory and antioxidant supplementation; ACE2 modulation strategies.

In summary, COVID-19 represents not only a respiratory and systemic challenge, but also a potentially chronic burden on skeletal health. Recognizing and addressing this emerging dimension is critical for holistic patient care in the post-pandemic era.
